# Safety and efficacy of SGLT2i administration in dilated cardiomyopathy: protocol for a systematic review and meta-analysis

**DOI:** 10.3389/fcvm.2025.1575493

**Published:** 2025-09-12

**Authors:** Ruolan Hu, Li Yu, Jinrong Li, Lei Liu, Yifei Li

**Affiliations:** Key Laboratory of Birth Defects and Related Diseases of Women and Children of MOE, Department of Pediatrics, West China Second University Hospital, Sichuan University, Chengdu, Sichuan, China

**Keywords:** SGLT2i, dilated cardiomyopathy, efficacy, outcomes, meta-analysis

## Abstract

**Background:**

Sodium-glucose co-transporter 2 inhibitors (SGLT2i) have emerged as a promising treatment for heart failure and cardiomyopathy. Furthermore, recent research has explored the use of SGLT2i in patients with dilated cardiomyopathy (DCM). However, the evidence that SGLT2i can improve left ventricular function and reduce symptoms of heart failure in DCM patients is limited.

**Objective:**

The objective of our study was to assess the efficacy and safety of SGLT2i in managing DCM patients with heart failure and to predict its effectiveness in DCM patients without heart failure. The benefits of SGLT2i alone or in combination will also be determined.

**Methods:**

A structured search of bibliographic databases (PubMed, Embase, and the Cochrane Central Register of Controlled Trials) will be undertaken to retrieve randomized controlled trials and cohorts that describe the efficacy and safety of SGLT2i as a major therapy strategy for DCM patients. To ensure that all relevant data were captured, the search did not contain any restrictions on language or publication time. Primary efficacy outcomes will be all-causes mortality and cardiovascular mortality. Primary safety outcomes will be the incidence of hypoglycemia, liver and renal injuries, and recurrent respiratory tract infections. After deduplication, citations will be screened independently by 2 authors, and selected for inclusion based on prespecified criteria. Data extraction and risk of bias assessment will be performed independently and in duplicate.

**Conclusions:**

This study could potentially provide new insights into the therapeutic strategies for dilated cardiomyopathy patients and reforming clinical guidelines for using SGLT2i to ensure patient safety and medicine efficacy.

**Trial Registration:**

PROSPERO CRD42023417892.

## 1 Introduction

Dilated cardiomyopathy (DCM) is characterized by myocardial weakening and ventricular enlargement, which can result in heart failure and other complications (1). Current DCM management entails a combination of pharmaceutical interventions, lifestyle modifications, and surgical interventions (2), which may ameliorate clinical outcomes. However, challenges remain in DCM therapy, as the underlying pathophysiological mechanisms of the disease are extremely complex (3), posing difficulties in developing targeted treatments. Although beta-blockers and angiotensin-converting enzyme inhibitors (ACEi) have been shown to alleviate symptoms and enhance outcomes, their efficacy is limited, and they may not be efficacious for all patients (4). Due to the heterogeneity of DCM, personalized treatment approaches that account for the individual patient's features and disease progression are essential. Furthermore, for patients with advanced DCM, such as those with refractory heart failure or severe symptoms, heart transplantation or mechanical circulatory support devices are recommended treatments. However, the practical application of heart transplantation is limited due to the scarcity of organ donors and the possible contraindications of patients (5).

Sodium-glucose co-transporter 2 inhibitors (SGLT2i) has emerged as a promising treatment for heart failure and cardiomyopathy, both of which are marked by the heart's ineffective blood pumping (6, 7). SGLT2i have been demonstrated to mitigate the risk of cardiovascular death in patients with heart failure and reduced ejection fraction (HFrEF) (8). Studies have further revealed that SGLT2i can improve the quality of life and alleviate symptoms of heart failure, including reducing hospitalizations and enhancing exercise capacity (9). In addition to their glucose-lowering effects, SGLT2i may offer cardioprotective benefits by reducing oxidative stress, inflammation, and fibrosis in the myocardium (10, 11). Furthermore, recent research has explored the use of SGLT2i in patients with DCM. These studies have reported that SGLT2i can enhance left ventricular function and alleviate heart failure symptoms in patients with DCM (12).

Although SGLT2i shows promise in the treatment of DCM, its use is limited by potential side effects, such as hypotension (13, 14), Euglycemic Diabetic Ketoacidosis (15) and genital infection (13, 14, 16), as demonstrated in the treatment of heart failure. Moreover, the limited evidence of SGLT2i in the treatment of DCM and the lack of personalized treatment methods make the application of SGLT2i in the treatment of DCM full of uncertain (17, 18). While some studies have shown that SGLT2i can improve left ventricular function and reduce heart failure symptoms in DCM patients, the current individual studies show conflicting results, so more research is needed to determine their long-term efficacy and safety in this population. Furthermore, SGLT2i may not be effective in patients with advanced DCM, who may require more aggressive treatment options such as mechanical circulatory support or heart transplantation. Therefore, a systematic review and meta-analysis are planned to assess the efficacy and safety of SGLT2i in the treatment of DCM with heart failure and to predict whether SGLT2i would benefit patients with DCM without heart failure. The review aims to identify the benefits of SGLT2i as solo or combination therapy and provide new insights into the therapeutic strategies for DCM.

## 2 Methods

This systematic review protocol is prepared according to the Preferred Reporting Items for Systematic Reviews and Meta-Analysis Protocols (PRISMA-P) statement (19). This review protocol has been registered in the PROSPERO International Prospective Register of Systematic Reviews (CRD42023417892). As this is a systematic literature research, ethical approval is waived.

### 2.1 Eligibility criteria

#### 2.1.1 Inclusion criteria

• Population: The patients have been diagnosed as DCM with imaging results, and the heart function (including HFpEF, HFmrEF, and HFrEF) should be addressed;
• Intervention: Studies that describe the administration of any amount and any type of SGLT2i as a major therapy strategy for participants who have confirmed DCM diagnosis. Studies that describe the coadministration of other traditional treatments for DCM with SGLT2i will also be included with clear statements of therapeutic formula formation; Device implantation will also be taken into consideration;
• Comparator: For inclusion, studies must contain a comparator group of DCM patients who have received other strategies for disease treatment or who have received a placebo. Studies that compare different SGLT2i dosing schemes and no placebo, will also be considered;
• Setting: Studies will not be restricted to location or setting. All circumstances where SGLT2i was administered to DCM will be examined;
• Types of study to be included: Prospective and retrospective studies will both be included; Cohort research and RCTs will also be considered;
• Outcomes: The primary efficacy outcomes will include risk ratio of all-causes death; cardiac-related risk ratio; and the secondary outcomes include cardiac function, severity of heart failure; time of hospitalization, heart transplantation risk ratio, device implantation risk ratio; arrhythmia attacks; serum level of cardiac troponin I(cTnI) and brain natriuretic peptide(BNP). The safety evaluations will include the incidence of hypoglycemia; liver and renal injuries; recurrent respiratory tract infections; incidence of genital and urinary tract infections; hypotension and dehydration; diabetic ketoacidosis; and bone fractures.

#### 2.1.2 Exclusion criteria

The exclusion criteria include: (1) Animal studies, case reports, case series analysis, and review articles should be excluded; (2) Overlapping positive genetic variants of hypertrophic and arrhythmogenic cardiomyopathy; (3) Definitely diagnosed as diabetic cardiomyopathy; 4) Suspected myocarditis.

### 2.2 Search strategy

We will search PubMed, Embase, Web of Science, Scopus, the ClinicalTrials.gov registry and the Cochrane Central Register of Controlled Trials will be searched using the MeSH terms “SGLT2i”, “SGLT2 inhibitor”, “sodium-glucose cotransporter 2 inhibitor”, “DCM”, “dilated cardiomyopathy”, “empagliflozin”, “dapagliflozin” and “sotagliflozin”. The search strategy is developed by the research groups and adjusted several times in attempts to reach a search strategy with high sensitivity and specificity following the objective of the research. The search strategy is presented in the online Supplementary Table S1.

### 2.3 Study selection

Citations will be collected in a reference manager software program (EndNote X9) and duplicates will be eliminated. Two authors (HR and YL) will review all the included studies independently by assessing the titles and abstracts after duplication removal. The full text will be further reviewed for inclusion. Finally, the final included studies will be cross-checked to ensure they meet the criteria. Disagreements will be resolved by discussion leading to a consensus and involvement of the third author (LY) if necessary. We will record reasons for the exclusion of studies analyzed in full text.

### 2.4 Data extraction and analysis

A standard data collection will be performed among the included studies. The two reviewers (HR and YL) will independently extract the data from the included studies and fill out the data collection form. Differences and uncertainties will be resolved through consensus between the two reviewers or by requiring the third author (LY) to make a final decision. The following data will be extracted and organized:

• General information: First author; Article title; Publication journal; Year of publication; Countries the major population enrolled;
• Methods: Study design; Control setting; Whether randomization design involved; Distribution concealment method; Blind method; The inclusion criteria and exclusion criteria of enrolled population; Echocardiographic assessment; Cardiac MRI measurement; The protocol for whole exome sequencing(WES) analysis; Family history;
• Subjects: Age; Gender; The severity of heart failure (AHA stage and NYHA classification); Time duration since DCM diagnosis; Results of genetic analysis; Race; Whether combined with arrhythmia;
• Intervention: Type of SGLT2i; Dose of SGLT2i, Course of treatment and duration of treatment; Combination of other medication; Whether received device implantation;
• Results: Survival rate; Cardiac function; Structure of biventricles; Level of cTnI and BNP; Changes of arrhythmia; Follow-up duration; Ratio of heart transplantation; Pacemaker or implantable cardioverter defibrillator (ICD) implantation; Treatment costs; Complications.

### 2.5 Assessment of risk of bias and quality

The risk of bias (ROB) of randomized controlled trials will be assessed by two independent reviewers using the ROB tool in the Cochrane Handbook 5.1.0. The indicators of ROB include random sequence generation, allocation concealment, blinding of patients and caregivers, blinding of outcome assessment, data completeness, selective outcome reporting, and other biases. Each indicator will be judged as high risk, low risk, or unclear of risk for the result of the evaluation. Different opinions on the evaluation indicators will be resolved through discussion with the third researcher. Newcastle–Ottawa Scale which has eight items was used to assess the quality of the cohort studies. One or two points will be awarded for each criterion and the points will be added up to compare study quality in a quantitative manner. Total points of <7 and ≥7 will assigned for low and high quality of studies, respectively. Two reviewers will carry out the assessment independently. Any disagreements will be resolved through discussion until a consensus is reached. All the researchers involved in study selection and quality assessment had been trained in the systematic review study program in Cochrane Center of China at West China Hospital, Sichuan University. GRADE approach will be used to rate the certainty of evidence for primary outcomes. Evidence from RCTs started as high certainty but was downgraded for limitations (e.g., allocation concealment failure), inconsistency (*I*^2^ > 60%), or imprecision (95% CI crossing the null effect).

### 2.6 Publication bias

When a meta-analysis includes 10 or more RCTs, we will assess asymmetry using funnel plots visually. Publication bias will be tested using funnel plots and Egger's test with Stata statistical software (STATA) version 16.0. An asymmetric distribution of data points in the funnel plot and a quantified result of *P* < 0.05 in the Egger's test indicates the presence of potential publication bias.

### 2.7 Heterogeneity

The *χ*^2^ test will be used to examine heterogeneity in pooling sensitivity and specificity; the statistical significance level will be set at 0.1. The I square (*I*^2^) test will also be conducted in every pooling analysis to quantitatively estimate the proportion of total variation across studies that is attributable to heterogeneity rather than chance. The values of *I*^2^ will be considered as follows: 0%–30% might not be important, 30%–50% may represent moderate heterogeneity, 50%–75% may represent substantial heterogeneity and 75%–100% represents considerable heterogeneity.

### 2.8 Subgroup analysis

Subgroup analysis will be conducted to explore potential sources of heterogeneity. We will perform subgroup analysis for the type of control group (placebo and standard treatment, or more detailed subgroups such as beta-blockers, ACEi, and mineralocorticoid receptor antagonist), SGLT2i (Sanagliflozin, Dapagliflozin and Empagliflozin), age categories (under 18 years and over 18 years), types of heart failure (HFpEF, HFmrEF, and HFrEF), whether administrate combination medication therapy, whether apply assistant device implantation (pacemaker, left ventricular assistant device, ECMO, and ICD). If possible, subgroup analyses will also be performed according to age, gender, and country area. We will consider meta-regression when there are more than ten studies.

### 2.9 Sensitivity analysis

To determine whether any single study incurred undue weight in the analysis, a sensitivity analysis will be conducted for every study using STATA 16.0 for the meta-analysis fixed/random-effects estimates.

### 2.10 Statistical analysis

Data analysis will take the form of a narrative synthesis in which studies are described and categorized according to design and purpose. A meta-analysis will be conducted if effect measures for individual studies are provided or can be calculated and if studies are suitably homogeneous. The STATA 16.0 software will be used for data analysis and synthesis. Continuous variables will be expressed in Mean Difference (MD)/Standard Mean Difference (SMD) with 95% confidence intervals (CIs), and categorical variables will be expressed in Risk Ratio (RR) with 95% CIs. According to the differences in the age, sex, and nationality of the population and the different lengths and doses of SGLT2i, we will use the random effect model to analyze the results and calculate the outcomes using the inverse variance method.

## 3 Results

The outcome measures will include the risk ratio of all-causes dead and cardiac-related, as well as the incidence of other organ damage among patients diagnosed with DCM. We will regularly search and write the systematic review when there is a certain number of clinical studies (6–8). Finally, the results will be published in a peer-reviewed journal and on PROSPERO.

## 4 Discussion

The increasing number of articles confirmed its advantages in managing heart failure. However, the experiences of SGLT2i in cardiomyopathy, especially for inherited cardiomyopathy, were very limited. There was no guideline for dilated cardiomyopathy stating the importance of SGLT2i in the management. Animal studies have confirmed that dapagliflozin can improve cardiac function in DCM by reducing TLR4 expression and inhibiting NLRP3 inflammasome activation (20). There are also a few observational studies on DCM, most of which suggest that SGLT2i combined with routine therapy is beneficial to cardiac function, but whether it is the result of SGLT2i alone is still doubtful.

Most clinical studies have focused on patients with non-ischemic dilated cardiomyopathy (NIDCM) (LVEF ≤ 40%). SGLT2i [dapagliflozin (16)/empagliflozin (21)] clearly reduces the risk of hospitalization for heart failure in patients with DCM, possibly by improving myocardial energy metabolism and alleviating fibrosis. The results of Hong et al. are consistent with those of McMurray et al. except that the population he included was not associated with diabetes (22). For another study on non-diabetic DCM patients, empagliflozin significantly improved cardiac function, biomarkers, and exercise tolerance with a good safety profile (23). A meta-analysis showed an interesting result because guidelines strongly recommend ICD implantation in NIDCM patients, and the article found that the addition of ARNi and SGLT2i did not affect mortality for primary prevention of ICD in NIDCM patients (24). Therefore, it is still unclear whether SGLT2i is really better than traditional treatment in patients with DCM, and more clinical studies are needed to confirm this. The application of SGLT2i in heart failure has been relatively mature, and clinical articles have shown that SGLT2i is effective in the treatment of heart failure complicated with dilated cardiomyopathy. Based on the homology of pathological mechanisms, it is inferred that SGLT2i has effective metabolic regulation (such as increased utilization of ketone bodies), anti-remodeling (such as reduction of fibrosis), and cytoprotection (such as reduction of oxidative stress) in heart failure. We believe that the results of the analysis of SGLT2i efficacy in DCM patients with heart failure may extend to DCM patients without heart failure. And provide opportunities for early intervention. We expect a lower risk of adverse cardiovascular events and death and better cardiac function in the SGLT2i group than in the control group.

The main limitation of this study could be a high heterogeneity of included studies and patient cohorts, which may lead to reduced statistical power and the lack of generalizability of the results. The second point is the small number of included studies with small numbers. Subgroup analyses were planned to reduce heterogeneity, and careful textual analyses were planned for outcomes that could not be subjected to meta-analysis.

To the best of our knowledge, this is the first study to focus specifically on the effects of SGLT2i in patients with DCM. Although SGLT2i is widely used in cardiovascular diseases, there is a lack of evidence that it is effective on cardiac function in patients with DCM. This protocol can provide definite evidence regarding the efficacy and safety of SGLT2i for individuals affected by DCM. Moreover, these results may also provide evidence for guideline medication of DCM, and offer new effective treatment for DCM patients and improve their quality of life. With the results of this study, we hope to contribute to the growing body of evidence that SGLT2i is an exciting medicine, which may provide novel benefits to DCM patients.
