# Supplementary material for: Safety and efficacy of SGLT2i administration in dilated cardiomyopathy: protocol for a systematic review and meta-analysis
Source: Front Cardiovasc Med. 2025 Sep 12;12:1575493. doi: 10.3389/fcvm.2025.1575493 (PMC12463906; doi:10.3389/fcvm.2025.1575493)
Supplement: Supplementary file 1 [file Datasheet1.pdf]

Supplementary Material

Supplementary Table 1 Search strategy for Medline (via PubMed).

Number	Search items
1	sodium-glucose cotransporter 2 inhibitor [Title/Abstract]
2	SGLT2i [Title/Abstract]
3	SGLT2 inhibitor [Title/Abstract]
4	Sotagliflozin [Title/Abstract]
5	Dapagliflozin [Title/Abstract]
6	Empagliflozin [Title/Abstract]
7	1 or 2 or 3 or 4 or 5 or 6
8	Cardiomyopathy, Dilated [MeSH Terms]
9	dilated cardiomyopathy [Title/Abstract]
10	DCM [Title/Abstract]
11	dilated cardiomyopathies [Title/Abstract]
12	Familial Idiopathic Cardiomyopathies [Title/Abstract]
13	Familial Idiopathic Cardiomyopathy [Title/Abstract]
14	Congestive Cardiomyopathy [Title/Abstract]
15	Congestive Cardiomyopathies [Title/Abstract]
16	Idiopathic Dilated Cardiomyopathies [Title/Abstract]
17	Idiopathic Dilated Cardiomyopathy [Title/Abstract]
18	8 or 9 or 10 or 11 or 12 or 13 or 14 or 15 or 16 or 17
19	Randomized Controlled Trial [Article Type]
20	Clinical Trial [Article Type]
21	19 or 20
22	7 and 18 and 21

Supplementary Table 2 Search strategy for Embase.	
Number	Search items
#1	‘sodium-glucose cotransporter 2 inhibitor’/exp
#2	(SGLT2i OR SGLT2 inhibitor OR canagliflozin OR dapagliflozin OR empagliflozin OR ipragliflozin OR ertugliflozin): ti,ab
#3	#1 OR #2
#4	‘congestive cardiomyopathy’ /exp
#5	(congestive cardiomyopathies OR Dilated cardiomyopathy* OR DCM OR Familial Idiopathic Cardiomyopath* OR Idiopathic Dilated Cardiomyopath*): ti,ab
#6	#5 OR #6
#7	randomized controlled trial/exp
#8	'randomized controlled trial (topic)'/exp
#9	random*: ti,ab(random* OR randomized OR RCT OR placebo): ti,ab
#10	#7 OR #8 OR #9
#11	#3 OR #6 OR #10

Supplementary Table 3 Search strategy for Cochrane Central Register of Controlled Trials

Number	Search items
1	sodium-glucose cotransporter 2 inhibitor: ti,ab,kw
2	SGLT2i: ti,ab,kw
3	SGLT2 inhibitor: ti,ab,kw
4	(canagliflozin OR dapagliflozin OR empagliflozin OR ipragliflozin OR ertugliflozin): ti,ab,kw
5	1 OR 2 OR 3 OR 4
6	MeSH descriptor: [Cardiomyopathy, Dilated] explode all trees
7	dilated cardiomyopathy*: ti,ab,kw
8	congestive cardiomyopath*: ti,ab,kw
9	(DCM OR Familial Idiopathic Cardiomyopath* OR Idiopathic Dilated Cardiomyopath*): ti,ab,kw
10	6 OR 7 OR 8 OR 9
11	MeSH descriptor: [Randomized Controlled Trial] explode all trees
12	MeSH descriptor: [Randomized Controlled Trials as Topic] explode all trees
13	randomized. ti,ab,kw
14	placebo. ti,ab,kw
15	randomly ti,ab,kw
16	11 OR 12 OR 13 OR 14 OR 15
17	5 OR 10 OR 16
